# Development of a Single Construct System for Site-Directed RNA Editing Using MS2-ADAR

**DOI:** 10.3390/ijms21144943

**Published:** 2020-07-13

**Authors:** Tetsuto Tohama, Matomo Sakari, Toshifumi Tsukahara

**Affiliations:** Area of Bioscience and Biotechnology, Japan Advanced Institute of Science and Technology, 1-1 Asahidai, Nomi, Ishikawa 923-1292, Japan; s1850004@jaist.ac.jp (T.T.); m-sakari@jaist.ac.jp (M.S.)

**Keywords:** RNA editing, site-directed RNA editing, adenosine deaminases acting on RNA, MS2 system, single construct, MS2-ADAR

## Abstract

Site-directed RNA editing (SDRE) technologies have great potential for treating genetic diseases caused by point mutations. Our group and other researchers have developed SDRE methods utilizing adenosine deaminases acting on RNA (ADARs) and guide RNAs recruiting ADARs to target RNAs bearing point mutations. In general, efficient SDRE relies on introducing numerous guide RNAs relative to target genes. However, achieving a large ratio is not possible for gene therapy applications. In order to achieve a realistic ratio, we herein developed a system that can introduce an equal number of genes and guide RNAs into cultured cells using a fusion protein comprising an ADAR fragment and a plasmid vector containing one copy of each gene on a single construct. We transfected the single construct into HEK293T cells and achieved relatively high efficiency (up to 42%). The results demonstrate that efficient SDRE is possible when the copy number is similar for all three factors (target gene, guide RNA, and ADAR enzyme). This method is expected to be capable of highly efficient gene repair *in vivo*, making it applicable for gene therapy.

## 1. Introduction

Genome editing technologies such as the clustered regularly interspaced short palindromic repeat (CRISPR) and CRISPR-associated proteins (Cas) system are expected to provide novel therapeutics for a range of diseases [1,2,3]. Genome editing relies on the production of site-specific double-strand DNA breaks (DSBs) and subsequent endogenous repair through error-prone non-homologous end-joining (NHEJ) or error-free homology-directed repair (HDR) pathways [3,4,5]. Recently, DNA editing technologies have been developed that are based on different strategies [6,7,8]. These new approaches can edit a specific DNA base in a genome using an enzyme that deaminates adenosine or cytidine. Because these methods do not include a DSB step that results in small insertion or deletion mutations bridging the break site [9,10,11], they are predicted to be safer than conventional genome editing technologies for gene therapy applications.

However, all DNA editing technologies, including those mentioned above, suffer from problems that hinder gene therapy applications. Notably, errors (off-target mutations) in genomic DNA caused by DNA editing remain permanently in treated cells due to the persistence of DNA, and they can cause cell death or oncogenic transformation [9,12]. For this reason, cells harboring errors must be removed from patients to avoid permanent editing of the genome, but it is extremely difficult to selectively remove such cells using current technologies.

Instead of DNA editing, in the present work we decided to edit disease-related genes at the RNA level. When targeting RNA, a nucleotide change is transient [13,14,15]; hence, off-target mutations in RNA are also transient and are relatively harmless compared with mutations in the genome. Therefore, the effects of off-target mutations in RNA are likely to be far less damaging to cells, and removal of cells harboring mutated RNA is not likely to be necessary.

To achieve gene editing at the RNA level, we developed a site-directed RNA editing (SDRE) technology based on adenosine deaminases acting on the RNA deaminase domain (ADAR DD) and phage-derived MS2 (MS2-ADAR) system [16,17,18]. MS2-ADAR can alter RNA by converting specific adenosine (A) bases to inosine (I; recognized as G during translation). Some other SDRE approaches have been reported that utilize ADAR with a SNAP-tag [19], λN protein [20], and Cas13 [21]. These technologies use different factors to achieve SDRE, but the basic principles are similar for all. In recent years, these methods have been applied in vivo to treat diseases in mice [22], and the use of endogenous ADAR [23,24] and the development of cytidine (C) to uridine (U) SDRE technology [25] have also been reported.

Although many studies on SDRE have been carried out, most rely on introducing numerous copies of genes responsible for SDRE (especially guide RNA expression genes) relative to target genes. Our ultimate goal is gene therapy for patients suffering from genetic diseases, and achieving such a large ratio is not compatible with clinical applications. For example, in a clinical trial on gene delivery of a functional dystrophin transgene to skeletal muscle in patients with Duchenne’s muscular dystrophy using adeno-associated virus (AAV), the delivery efficiency was 0.01 to 2.56 vector copies per diploid genome [26]. In another example of clinical gene therapy targeting lipoprotein lipase deficiency, the delivery efficiency was undetectable up to 14.4 (average = 3.2) vector copies per diploid genome [27]. Although the delivery efficiencies in such trials cannot be directly compared due to differences in conditions (e.g., dose, AAV types, tissues), it is clearly not easy to stably introduce genes at copy numbers tens of times higher than those of target genes in patient tissues. For this reason, the ratio of the three main factors (genes encoding guide RNAs, fusion proteins containing ADARs, and target genes) should be explored. However, to our knowledge, studies on the ratio of these three factors in SDRE have not yet been reported.

Herein, we report the development of a system that can introduce similar copy numbers of genes encoding guide RNAs, fusion proteins, and target genes (EGFP reporter) into cultured cells using a plasmid vector containing one copy of each gene in a single construct. We employed a guide RNA that includes six copies of the MS2 RNA driven by the cytomegalovirus immediate-early 94 (CMV IE94) promoter described in our previous studies [16,17,18]. Some other research groups have reported higher editing efficiencies using guides in which MS2 RNAs or boxB RNAs bind to fusion proteins are present on both sides of antisense RNAs driven by the U6 promoter [22,28]. Therefore, in the present study we have attempted to use similar guide RNAs. Furthermore, by applying a long flexible linker to the fusion protein and reducing the size of the MCP, the editing efficiency was dramatically improved from our previous reports. By using this improved MS2-ADAR with a single construct system, the applicability of MS2-ADAR when the copy number is similar has been verified. We also compared the editing efficiencies of three types of MS2 coat proteins (MCPs), the components of MS2-ADAR, and verified the usefulness of adding a nuclear localization signal (NLS) to the fusion protein for SDRE at nucleus. Finally, we investigated whether MS2-ADAR could edit the mRNA of endogenous genes to demonstrate that it can also be applied to genes that could be targets for actual gene therapy.

## 2. Results

### 2.1. System for Introducing an Equal Number of Copies of the Three Factors

Our previously developed MS2-ADAR [16] did not have high editing efficiency (~5%), and further improvement is required for its application to gene therapy. We reviewed the components of conventional MS2-ADAR and made three major improvements. The first is the change from the conventional guide RNA that includes six copies of the MS2 RNA driven by CMV IE94 promoter to a guide RNA in which MS2 RNAs are present on both sides of antisense RNAs driven by the U6 promoter, which has been reported to have high SDRE efficiency [22,28]. The second is a change from the conventional direct connection between MCP and ADAR1 DD to the connection via a flexible linker called XTEN linker. In recent years, the XTEN linker has often been used as a linker for fusion proteins, and it has been reported that it may contribute to the protein stability [29]. In addition, in the study of the activity of fusions of catalytically inactive Cas9 and *Fok*I nuclease, the one with an XTEN linker had the highest activity [30], so we considered that it was a linker that could achieve high editing efficiency, and we applied the XTEN linker. The third is the application of only the part of MCP considered necessary from the conventional MCP to reduce the size of the fusion protein and increase its applicability to gene therapy. We verified the utility of the improved MS2-ADAR under more realistic conditions.

In order to perform SDRE under more realistic conditions, the copy number ratios of the three factors (guide RNA, fusion protein MCP-ADAR1 DD, and target gene) had to be equal in all transfected cells. In the conventional method introducing the three factors by separate plasmid vectors, even if each plasmid is transfected in an equal molar ratio, each plasmid is not uniformly introduced into each cell. Therefore, in the above method, the interpretation of the result may be difficult. Instead, we used a single construct containing a copy of each of the genes, so that the copy number ratios of the three factors were equal in cell units.

For the SDRE target, we used enhanced green fluorescent protein (EGFP) W58X bearing a nonsense mutation in the codon for tryptophan 58 (TGG) that turns it into a stop codon (TAG). Since full-length EGFP is not synthesized due to the mutation, EGFP W58X does not emit green fluorescence. If this stop codon (TAG) is repaired by A-to-I editing (I is recognized as G during translation) to regenerate the tryptophan (TGG) codon, full-length EGFP is synthesized, and green fluorescence should be observed.

**Figure 1:**
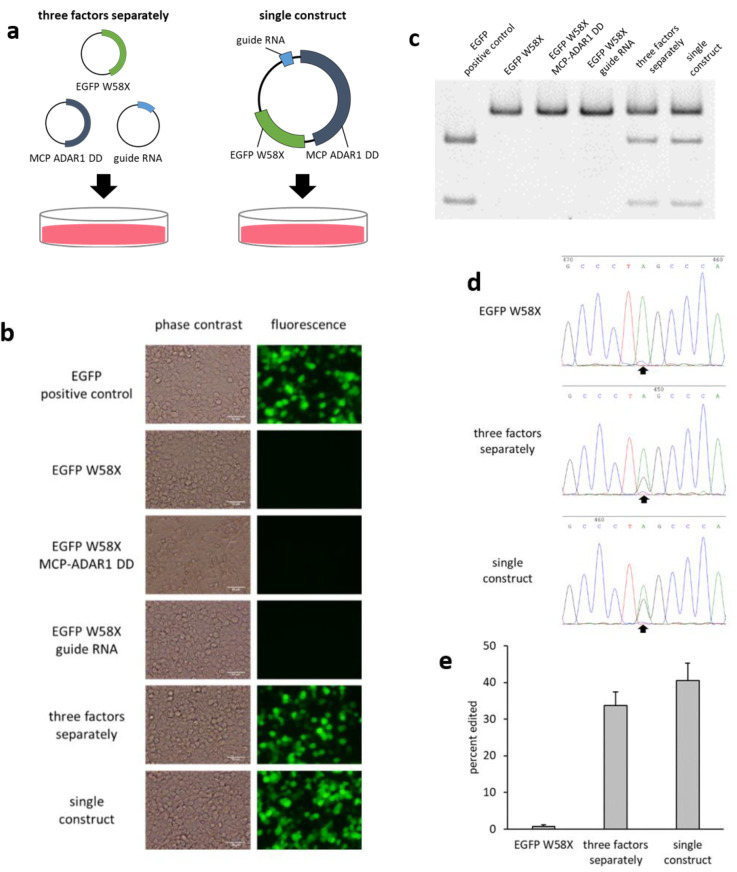
(**a**) The three factors were transfected into HEK293T cells using three separate plasmids each carrying a separate gene, or a single plasmid containing all three factors (single construct). (**b**) Fluorescence micrographs of HEK293T were obtained at 48 h after transfection (just before RNA collection). The images on the left are phase contrast images, and those on the right are fluorescence images. Scale bar = 50 µm. (**c**) At 48 h after transfection, RT-PCR products were digested by *Hae*III, and restriction fragment length polymorphism (RFLP) analysis was performed. (**d**) Waveform data around the target site from Sanger sequencing. Black arrows indicate the target base for EGFP W58X. (**e**) Bar graph showing the editing efficiency (%) calculated from the sequencing results using the peak height ratio method. Error bars represent standard error (*n* = 3).

After transfecting HEK293T cells with EGFP W58X, MCP-ADAR1 DD (fusion protein), and guide RNA using three separate plasmids (each carrying a different factor), or transfecting with a single construct carrying all three factors (Figure 1a), we carried out fluorescence microscopy. Green fluorescence was observed both when the three factors were transfected separately and when a single construct was used (Figure 1b). However, when transfecting with EGFP W58X, EGFP W58X and MCP-ADAR1 DD, or EGFP W58X and guide RNA only, green fluorescence was not observed.

In order to verify whether editing of the target site occurred at the RNA level, at 48 h after transfection total RNA was collected from cells, the target region was amplified by RT-PCR to generate a 329 bp amplicon, and restriction fragment length polymorphism (RFLP) analysis was performed to detect the single base difference. If SDRE occurred, the 329 bp fragment was cut into 228 bp and 101 bp fragments by the restriction enzyme *Hae*III. Cleavage of the fragment was detected both when the three factors were introduced separately and when a single construct was used (Figure 1c). However, when only EGFP W58X, EGFP W58X and MCP-ADAR1 DD, or EGFP W58X and guide RNA were transfected, cleavage was not observed.

To determine the editing efficiency, Sanger sequencing of RT-PCR products was performed (Figure 1d), and the editing efficiency was calculated from the peak height ratio [31]. At 48 h after transfecting the three factors separately, the editing efficiency was 34%, compared with 41% when transfecting with a single construct (Figure 1e).

### 2.2. Comparison of the Three Types of MCPs

SDRE using MS2-ADAR is dependent on MCP binding to MS2 RNA. Therefore, the binding affinity of MCP to the RNA might affect the editing efficiency. MCP harboring the N55K mutation at amino acid position 55 (MCP N55K) has higher binding affinity to MS2 RNA (a lower Kd for MCP-MS2 RNA interaction) than wild-type MCP (MCP WT) [32]; hence, we explored the capability of the mutant. In addition, we tested duplicated MCPs, which are known to behave like the WT protein [33]. In this study, duplicated MCPs bearing N55K mutations on each MCP (2MCP N55K) were used according to previous reports [34,35].

**Figure 2:**
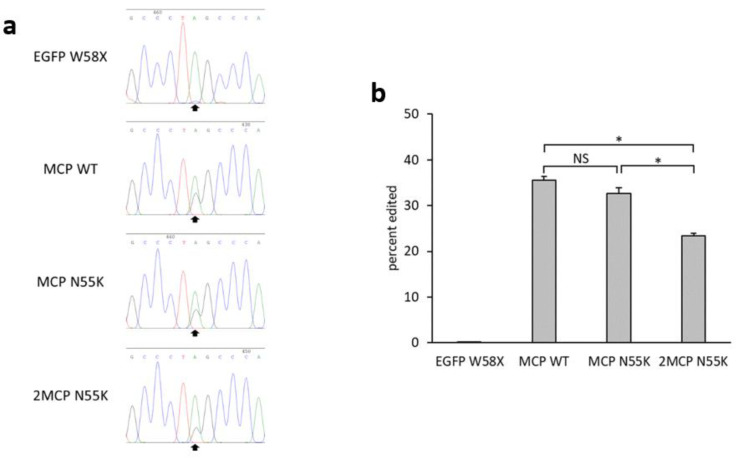
(**a**) Waveform data around the target site from Sanger sequencing. Black arrows indicate the target base for EGFP W58X. (**b**) Bar graph showing the editing efficiency (%) calculated from the sequencing results using the peak height ratio method. Error bars represent standard error (*n* = 3). Asterisks indicate statistically significant difference (*p* < 0.05). NS (not significant) signifies no statistically significant difference.

These three MCPs (MCP WT, MCP N55K, and 2MCP N55K) were compared using single constructs. The editing efficiencies of MCP WT, MCP N55K, and 2MCP N55K after 48 h were 35%, 33%, and 23%, respectively (Figure 2a,b).

### 2.3. Nuclear Localization of MCP-ADAR1 DD

A nuclear localization signal (NLS) is a short stretch of amino acids that mediates the transport of nuclear proteins into the nucleus [36,37]. MCP-ADAR1 DD has no NLS or other signals related to protein transport, indicating that it should be located in the cytoplasm. Addition of an NLS to MCP-ADAR1 DD should localize it to the nucleus and enable SDRE in the nucleus.

**Figure 3:**
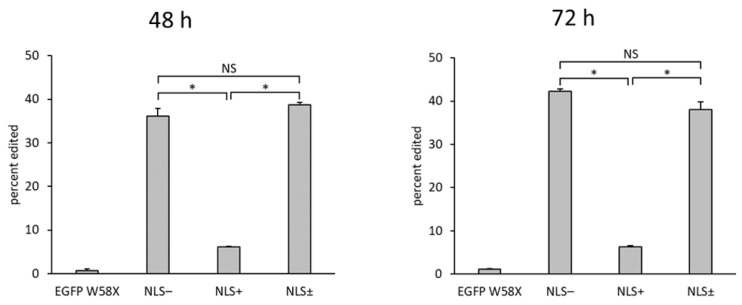
Bar graphs showing the editing efficiency (%) at 48 h (left) or 72 h (right) after transfection, calculated from the sequencing results using the peak height ratio method. Error bars represent standard error (*n* = 3). Asterisks indicate statistically significant differences (*p* < 0.05). NS (not significant) signifies no statistically significant difference.

In order to investigate the editing efficiency in the nucleus under conditions in which the copy number of the three factors is equal, we added an NLS to MCP N55K-ADAR1 DD in the single construct (NLS+). In addition to MCP N55K-ADAR1 DD (without NLS; NLS-) and NLS+, NLS- and NLS+ were co-transfected at the same molar ratio to validate the utility of simultaneous editing in the cytoplasm and nucleus (NLS±). The editing efficiencies of NLS-, NLS+, and NLS± after 48 h were 36%, 6%, and 39%, respectively (Figure 3). The editing efficiencies after 72 h were 42%, 6%, and 38%, respectively.

### 2.4. Applicability of MS2-ADAR to Endogenous Gene Editing

**Figure 4:**
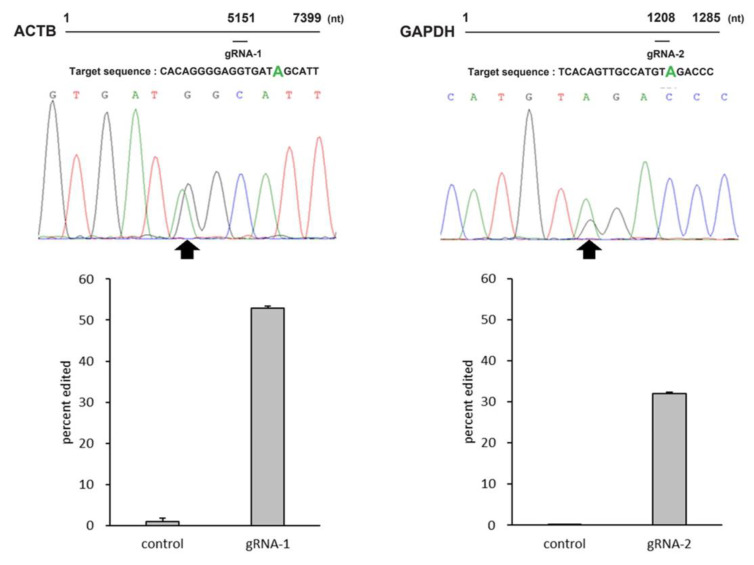
*ACTB* (left) and *GAPDH* (right) targeted base (green), the waveform data around the target site and bar graphs indicating the editing efficiencies. Black arrows indicate the target base for each gene. Error bars represent standard error (*n* = 3).

In order to demonstrate applicability of MS2-ADAR to gene therapy, we investigated whether it could edit endogenous genes that can be targets for actual gene therapy. Two endogenous genes (*ACTB*, *GAPDH*) were selected as candidates, and MCP N55K-ADAR1 DD and the guide RNA (gRNA-1 for *ACTB*, gRNA-2 for *GAPDH*) co-expressing plasmids were constructed and transfected into HEK293T cells. The editing efficiencies at ACTB and GAPDH targeting sites after 48 h were 53% and 32%, respectively (Figure 4).

## 3. Discussion

This study is the first to attempt SDRE under conditions in which the three main factors (target gene, guide RNA, and MCP-ADAR1 DD fusion protein) are present in an equal number of copies. Using a single construct containing a copy of each of the factors, we achieved relatively high efficiency (up to 42%). In addition, we showed MS2-ADAR worked not only on the reporter gene but also on several endogenous genes, suggesting its applicability to actual gene therapy.

In previous studies, expression levels of the guide RNA were extremely high relative to the target gene, which is far from ideal for gene therapy applications. In one recent study on SDRE using λN, although a very high editing efficiency (up to 57%) was achieved, the amount of guide RNA expression plasmid (U6 pENTR guide RNA vector) was 1.5 μg while that of the target gene (CFTR Y122X) expression plasmid was 25 ng, meaning that the guide RNA was 60-fold more abundant than the target gene [20]. In another study on SDRE using Cas13, the amount of guide RNA expression plasmid was 300 ng, while the amount of target gene expression plasmid (RNA editing reporter) was 40 ng (7.5-fold greater than the amount of guide RNA) [21]. It was previously unknown whether efficient editing can be achieved if the ratio of the three factors is the same, but we demonstrated that it is indeed possible in the present work. Thus, future SDRE applications employing systems other than MS2-ADAR should verify whether efficient editing is possible when the three factors are present in equal abundance.

We also compared the editing efficiencies of MCP WT, MCP N55K, and 2MCP N55K in MS2-ADAR. The editing efficiencies of MCP WT and MCP N55K were almost the same (35% and 33%, respectively; *p* = 0.27). This indicates that the binding affinity of MCP WT was sufficient for efficient editing by MS2-ADAR. The editing efficiency of 2MCP N55K was 23%, approximately 10% lower than that of MCP WT/N55K (*p* < 0.05). This reduced editing efficiency for 2MCP N55K might be because the two MCPs form a dimer that binds to MS2 RNA [38,39,40]. If two MCP-ADAR1 DDs also form a dimer at the MCP regions that, in turn, binds MS2 RNA, the MS2 RNA will recruit two ADAR1 DDs. However, since 2MCP N55K must contain two MCPs to form an intramolecular dimer [33], the MS2 RNA theoretically recruits only one ADAR1 DD. If this is the case, lower editing efficiency might occur due to fewer ADAR1 DD molecules being recruited in 2MCP-ADAR1 DD. Notably, if dimerization of MCP-ADAR1 DDs does indeed occur, it is a unique feature of MS2-ADAR not found in other SDRE technologies.

We verified the nuclear localization of MCP-ADAR1 DD by adding an NLS to the protein in the single construct. The editing efficiency of NLS+ was 6%, which was much lower than that of NLS- (*p* < 0.05). However, the efficiency of NLS+ was reported to be the same as that of NLS- [20]. NLS+ might have achieved the same editing efficiency as NLS- because the quantity of guide RNA introduced was much greater than that of the target gene, as explained above. If the editing efficiency of NLS+ drops significantly when the ratio of the three factors is equal, applying NLS+ to gene therapy would be difficult. Indeed, another study showed that NLS+ is significantly less efficient than NLS-, although a nuclear export signal was introduced for NLS- [22]; hence, careful judgment should be exercised when considering whether NLS is effective or not. SDRE utilizing an NLS is still in its infancy, and further study is needed to determine the usefulness of an NLS, especially in gene therapy applications.

In conclusion, we demonstrated that efficient editing can be achieved even under conditions in which the three main factors are present in equal abundance (equal copy number). Future studies are needed to determine whether highly efficient gene repair can be achieved *in vivo* for gene therapy applications.

## 4. Materials and Methods

### 4.1. Plasmid Construction

As a reporter gene of SDRE, a plasmid expressing EGFP W58X (TGG > TAG; A of TAG is the target base) based on backbone vector pcDNA3 (Addgene, MA, USA) was prepared by site-directed mutagenesis [16].

For the guide RNA, the CMV IE94 promoter of pCS2+ (Addgene) was replaced with the human U6 promoter (hU6 promoter), and the guide RNA was then inserted downstream of the hU6 promoter. The guide RNA sequence (5′-gaacatgaggatcacccatgtc**tgggccagggcacgggcagct**aacatgaggatcacccatgtctttt-3′), in which the MS2 RNA is underlined and bold letters indicate antisense RNA recognizing the target, was designed so that MS2 RNAs are on both sides of the antisense RNA to enable efficient SDRE [22,28].

Regarding the fusion protein comprising MCP and ADAR1 deaminase domain (ADAR1 DD) driven by the CMV IE94 promoter within the pCS2+MT vector (Addgene), MCP is at the N-terminus and ADAR1 DD is at the C-terminus. These two proteins were linked via a glycine–serine (GS) + XTEN linker (GGSGSGAGSGSSGSETPGTSESATPES). Wild-type MCP (MCP WT) was prepared by site-directed mutagenesis (AAG > AAC) by PCR using MS2-HB plasmid (Addgene) harboring the MCP N55K sequence. MCP N55K was prepared from MS2-HB. 2MCP N55K, comprising two copies of MCP N55K in tandem, was prepared by inverse PCR using the MCP N55K-ADAR1 DD construct as a template and inserting another MCP N55K fragment amplified by PCR. The amino acid sequence of 2MCP N55K is the same as that of the 2MCP N55K part of MS2-HB. ADAR1 DD was amplified by PCR from a plasmid used in our previous study [16] as a template.

For the single construct, the guide RNA expression gene and MCP-ADAR1 DD expression gene were inserted into the EGFP W58X expression plasmid using standard restriction enzyme cloning. In the single construct, the woodchuk hepatitis virus posttranscriptional regulatory element (WPRE) was inserted into the 3′-untranslated region (UTR) of MCP-ADAR1 DD.

To validate the utility of the NLS for SDRE, three copies of the NLS of the SV40 Large T Antigen (APKKKRKVDPKKKRKVDPKKKRKV) [20,41] were added in series to the N-terminus of MCP N55K-ADAR1 DD.

Plasmids used in this work were amplified using *Escherichia coli* DH5α competent cells (Takara Bio, Siga, Japan), then extracted using NucleoSpin Plasmid Transfection-grade (MACHEREY-NAGEL, Düren, Germany). The DNA concentration was measured using a NanoDrop 1000 Spectrophotometer (Thermo Fisher Scientific, MA, USA).

Detailed information for all plasmids used in reporter gene editing assay is included in Table S1.

### 4.2. Cell Culture

HEK293T cells from RIKEN BRC CELL BANK were maintained on 60 × 15 mm Falcon Cell Culture Dishes (Corning, NY, USA) in Dulbecco’s modified Eagle’s medium (Nacalai Tesque, Kyoto, Japan) supplemented with fetal bovine serum (Thermo Fisher Scientific) at a volume ratio of 10:1 DMEM:FBS under 5% CO_2_ at 37 °C. Cells were used in experiments after at least three passages from frozen stocks.

### 4.3. Transfection

At 16–24 h before transfection, HEK293T cells were seeded in Falcon 48-well Multiwell Cell Culture Plates (Corning) at a density of 3.9 × 10^5^ to 7.0 × 10^5^ cells/well. Before transfection, the medium was removed so that the volume per well was 0.15 mL, and 0.15 mL of fresh DMEM + FBS was added. Next, 25 µL of Opti-MEM (Thermo Fisher Scientific), 1.25 µL of 1.0 µg/µL PEI MAX (Polysciences, Illinois, USA), and 250 ng of plasmid DNA were mixed, incubated for 20 min, and added to each well. At 24 h after transfection, 0.18 mL of medium was removed from each well, and 0.18 mL of fresh DMEM + FBS was added. At 48 or 72 h after transfection, total RNA from transfected cells was collected immediately after green fluorescence was observed using a BZ-8000 fluorescence microscope (Keyence, Osaka, Japan).

Details of the conditions of reporter gene editing assay are included in Table S2.

### 4.4. Determination of Editing Efficiency

At 48 or 72 h after transfection, total RNA was extracted using TRIzol Reagent (Thermo Fisher Scientific) at 0.1 mL/well according to the manufacturer’s protocol. Extracted total RNA was treated with 1.5–5 units of Recombinant DNaseI (Takara Bio) for 30 min at 37 °C (total volume 10 µL), and 1 µL of 20 mM ethylenediaminetetraacetic acid (EDTA) was added and incubated for 10 min at 75 °C.

Using 300–500 ng of total RNA treated with DNase I, cDNA was synthesized with 50 units of ReverTra Ace (TOYOBO, Osaka, Japan) by incubating for 10 min at 50 °C, then incubating for 5 min at 99 °C. Primer No. 1 employed for cDNA synthesis is listed in Table S3.

PCR was performed with GoTaq Flexi DNA Polymerase (Promega, Wisconsin, USA) using 0.2–10 ng of cDNA (converted to amount of total RNA) as template to amplify the DNA region containing the target base in EGFP W58X. Primers No. 2 and No. 3 used for PCR are listed in Supplementary Table S3. In most replicates, using the above product as template, a second round of PCR was performed using primers No. 4 and No. 5 (Table S3). PCR products were purified using NucleoSpin Gel and PCR Clean-up (MACHEREY-NAGEL).

Sanger sequencing of purified samples was performed by Eurofins Genomics (Tokyo, Japan). For sequencing, a reverse primer was employed as described previously [42]; primer No. 6 (Table S3) was used for purified samples following PCR with No. 2 and No. 3, and No. 7 was used for purified samples following the second-round PCR with No. 4 and No. 5. Since the edited region comprises a mixed peak of T (unedited) and C (edited), the editing efficiency (%) was calculated by measuring the height of each peak as described previously [31].

### 4.5. RFLP Analysis

After cDNA synthesis using primer No. 8 (Table S3), nested PCR was performed to amplify a 329 bp DNA fragment containing the target base in EGFP W58X; first-round PCR was performed using primers No. 9 and No. 3 in Supplementary Table S3, then second-round PCR was performed using primers No. 4 and No. 5. The sequence of the region containing the target site before SDRE is 5′-… ctagcc…-3′, and the sequence after SDRE is 5′-…ctggcc…-3′. When SDRE occurs correctly, the 329 bp fragment is cut into 228 bp and 101 bp fragments by restriction enzyme *Hae*III, which recognizes and cuts the 5′-ggcc-3′ sequence. In order to detect target base conversion, *Hae*III (Takara Bio) digestion was performed for 1 h at 37 °C, and the presence or absence of fragment cleavage was observed by polyacrylamide gel electrophoresis. For nucleic acid gel staining, SYBR Green I (Lonza, Basel, Switzerland) was employed according to the manufacturer’s protocol.

### 4.6. Endogenous Gene Editing Assay

ACTB and GAPDH mRNA targeting guide RNAs (gRNA-1 and gRNA-2, respectively) were designed and constructed MCP N55K-ADAR1 DD and gRNA-1/gRNA-2 co-expressing plasmids. On the day before transfection, HEK293T cells were seeded in Falcon 12-well Multiwell Cell Culture Plates (Corning) at a density if 4.0 × 10^5^ cells/well. A total of 1.5 μg of each plasmid was transfected into the cells. At 48 h after transfection, editing efficiencies were determined in the same way as 4.7. Detailed information for plasmids used in this assay is included in Table S4.

### 4.7. Statistical Analysis

In all experiments for determining editing efficiency, three independent experiments were performed. Multiple comparisons were made by performing a paired *t*-test followed by the Bonferroni correction method.

## Supplementary Materials

Supplementary materials can be found at https://www.mdpi.com/1422-0067/21/14/4943/s1.
