# Supplementary material for: Development of a Single Construct System for Site-Directed RNA Editing Using MS2-ADAR
Source: Int J Mol Sci. 2020 Jul 13;21(14):4943. doi: 10.3390/ijms21144943 (PMC7404196; doi:10.3390/ijms21144943)
Supplement: Supplementary file 1 [file ijms-21-04943-s001.pdf]

Table S1. Plasmids used for reporter gene editing assay

Plasmid	Size (bp)	Description	Remarks	Sequence
pCS2+	4095	Backbone vector harboring the CMV IE94 promoter.	Negative control for MCP-ADAR1 DD.	https://benchling.com/s/seq-Ey8krl0WHYAWtxDJw1jh
pCS2+ U6	2470	Backbone vector in which the CMV IE94 promoter of pCS2+ is replaced with the hU6 promoter.	Negative control for guide RNA.	https://benchling.com/s/seq-4ER61fxe7jCedEYHZPsH
EGFP	3874	EGFP expression plasmid driven by the CMV promoter in pcDNA3.	The SV40 origin in the original pcDNA3 plasmid is deleted.	https://benchling.com/s/seq-L6ZL9aBuxZNNjN8wbxKK
EGFP W58X	3874	Plasmid carrying the introduced W58X mutation in EGFP.		https://benchling.com/s/seq-lEiXwWIRjtnfKYzbkJGp
MCP WT-ADAR1 DD	5799	MCP WT-ADAR1 DD expression plasmid driven by the CMV IE94 promoter in pCS2+MT.		https://benchling.com/s/seq-h7A7atghYgwHzU0rDEzj
guide RNA	2510	Guide RNA expression plasmid driven by the hU6 promoter in pCS2+ U6.		https://benchling.com/s/seq-ECFwFJhlk0iv3SdHQWdG
MCP WT-ADAR1 DD (SC)	7964	Plasmid expressing the three factors including MCP WT-ADAR1 DD.	SC stands for single construct.	https://benchling.com/s/seq-o4EgnNlbWGIv0JqgLPEz
MCP N55K-ADAR1 DD (SC)	7964	Plasmid expressing the three factors including MCP N55K-ADAR1 DD.		https://benchling.com/s/seq-v41rqDcooA9e5XkS27qj
2MCP N55K-ADAR1 DD (SC)	8360	Plasmid expressing the three factors including 2MCP N55K-ADAR1 DD.		https://benchling.com/s/seq-tJ0hakFu0OVwSBz8BEfc
NLS-MCP N55K-ADAR1 DD (SC)	8060	Plasmid expressing the three factors including NLS-MCP N55K-ADAR1 DD.		https://benchling.com/s/seq-SUTuuiak5eFvgEFmyqZE

Table S2. Experimental conditions in reporter gene editing assay

System for introducing an equal number of copies of the three factors

No.	Plasmids transfected
1	EGFP 30 ng, pCS2+ 100 ng, pCS2+ U6 120 ng
2	EGFP W58X 30 ng, pCS2+ 100 ng, pCS2+ U6 120 ng
3	EGFP W58X 30 ng, MCP WT-ADAR1 DD 100 ng, pCS2+ U6 120 ng
4	EGFP W58X 30 ng, pCS2+ 100 ng, guide RNA 120 ng
5	EGFP W58X 30 ng, MCP WT-ADAR1 DD 100 ng, guide RNA 120 ng
6	MCP WT-ADAR1 DD (SC) 250 ng

Comparison of the three types of MCPs

No.	Plasmids transfected
1	EGFP 250 ng
2	EGFP W58X 250 ng
3	MCP WT-ADAR1 DD (SC) 250 ng
4	MCP N55K-ADAR1 DD (SC) 250 ng
5	2MCP N55K-ADAR1 DD (SC) 250 ng

Nuclear localization of MCP-ADAR1 DD

No.	Plasmids transfected
1	EGFP 250 ng
2	EGFP W58X 250 ng
3	MCP N55K-ADAR1 DD (SC) 250 ng
4	NLS-MCP N55K-ADAR1 DD (SC) 250 ng
5	MCP N55K-ADAR1 DD (SC) 124 ng, NLS-MCP N55K-ADAR1 DD (SC) 126 ng

Table S3. Primers mainly used in this study

No.	Sequence (5′ > 3′)	Use
1	ctgatctagaggtaccggatcctttttttttttttttttt	cDNA synthesis
2	acccaagctatccatcacactg	PCR (forward primer)
3	ctgatctagaggtaccggatcc	PCR (reverse primer)
4	gccacaagttcagcgtgtc	PCR (forward primer)
5	gtcctccttgaagtcgatgc	PCR (reverse primer)
6	agcaggaccatgtgatc	sequencing
7	ccttgaagaagatggtg	sequencing
8	ctgatctagaggtaccggatccgtgacactatagaatagg	cDNA synthesis
9	atggtgagcaagggcgag	PCR (forward primer)

Sequences underlined in primers No. 1 and No. 8 indicate the adapter site where primer No. 3 anneals.

Table S4. Plasmids used for endogenous gene editing assay

Plasmid	Size (bp)	Description	Remarks	Sequence
MCP N55K-ADAR1 DD-gRNA-1	6124	Plasmid expressing MCP N55K-ADAR1 DD and gRNA-1	Editing ACTB mRNA	https://benchling.com/s/seq-2XvMSSBAh50Ebq9GfkGH
MCP N55K-ADAR1 DD-gRNA-2	6124	Plasmid expressing MCP N55K-ADAR1 DD and gRNA-2	Editing GAPDH mRNA	https://benchling.com/s/seq-8e3ELv4emZHD7lMcaswP
